# Galectin-9 Activates and Expands Human T-Helper 1 Cells

**DOI:** 10.1371/journal.pone.0065616

**Published:** 2013-05-31

**Authors:** Marloes J. M. Gooden, Valerie R. Wiersma, Douwe F. Samplonius, Jurjen Gerssen, Robert J. van Ginkel, Hans W. Nijman, Mitsuomi Hirashima, Toshiro Niki, Paul Eggleton, Wijnand Helfrich, Edwin Bremer

**Affiliations:** 1 Department of Surgery, Translational Surgical Oncology, University Medical Center Groningen (UMCG), University of Groningen, Groningen, The Netherlands; 2 Department of Obstetrics and Gynecology, University Medical Center Groningen (UMCG), University of Groningen, Groningen, The Netherlands; 3 GalPharma Co., Ltd., Kagawa, Japan; 4 Department of Immunology and Immunopathology, Kagawa University Faculty of Medicine, Kagawa, Japan; 5 University of Exeter Medical School, Exeter, United Kingdom; University of Oslo, Norway

## Abstract

Galectin-9 (Gal-9) is known for induction of apoptosis in IFN-γ and IL-17 producing T-cells and amelioration of autoimmunity in murine models. On the other hand, Gal-9 induced IFN-γ positive T-cells in a sarcoma mouse model and in food allergy, suggesting that Gal-9 can have diametric effects on T-cell immunity. Here, we aimed to delineate the immunomodulatory effect of Gal-9 on human resting and *ex vivo* activated peripheral blood lymphocytes. Treatment of resting lymphocytes with low concentrations of Gal-9 (5–30 nM) induced apoptosis in ∼60% of T-cells after 1 day, but activated the surviving T-cells. These viable T-cells started to expand after 4 days with up to 6 cell divisions by day 7 and an associated shift from naïve towards central memory and IFN-γ producing phenotype. In the presence of T-cell activation signals (anti-CD3/IL-2) Gal-9 did not induce T-cell expansion, but shifted the CD4/CD8 balance towards a CD4-dominated T-cell response. Thus, Gal-9 activates resting T-cells in the absence of typical T-cell activating signals and promotes their transition to a T_H1/C1_ phenotype. In the presence of T-cell activating signals T-cell immunity is directed towards a CD4-driven response by Gal-9. Thus, Gal-9 may specifically enhance reactive immunological memory.

## Introduction

The galectin family is a group of glycan-binding proteins characterized by conserved carbohydrate recognition domains (CRDs) that bind glycosylated proteins. Galectins are involved in various processes including embryonic development, tumor biology and regulation of the immune system [1]. Within this family, Galectin-9 (Gal-9) has gained attention as a multifaceted player in adaptive and innate immunity, in particular in T-cell development and homeostasis [2]. The most prominent effects reported for Gal-9 are the induction of apoptosis in subsets of differentiated T-cells, particularly in CD4^+^ T-helper 1 (T_H1_) and T-helper 17 (T_H17_) cells [3], [4], [5], [6], [7], and a stimulatory effect on regulatory T-cell (T_reg_) activity [6], [8]. In view of these immunomodulatory effects, Gal-9 has been tested as a potential therapeutic agent for various autoimmune diseases. Treatment with Gal-9 ameliorated disease in mouse models of experimental autoimmune encephalomyelitis [3], arthritis [9] and diabetes [10], [11], by reducing the number of autoreactive T_H1_ and T_H17_ cells and decreasing circulating IFN-γ concentrations. In contrast, treatment with Gal-9 stimulated anti-tumor T-cell immune responses in a sarcoma bearing mouse model [12]. Here, recombinant Gal-9 induced cytotoxic T-cells (CTLs) and increased IFN-γ concentrations. In addition, in a recent study focused on food-allergy treatment of *ex vivo* activated human T-cells with Gal-9 promoted T_H1_ generation as well as IFN-γ production [13]. These data imply that Gal-9 can have a Janus-like dual activity; inhibiting immunity in autoimmune disease on the one side and stimulating immunity in cancer and allergy on the other side.

The immunomodulatory effects of Gal-9 were initially attributed to signaling via T-cell immunoglobulin and mucin domain-3 (TIM-3) [3], a prominent T-cell inhibitory receptor and a marker for T-cell exhaustion that is currently being evaluated as a target for antibody-based therapy in cancer [14]. However, it has become clear that, aside from TIM-3, Gal-9 can signal via other receptors on T-cells [15], like protein disulfide isomerase [16], [17], CD40 [18] and possibly other, yet unidentified receptors. Indeed, the outcome of Gal-9 signaling on T-cells likely depends on the specific receptor being activated by Gal-9 as well as the presence of additional (T-cell) skewing stimuli. In this respect, most experimental murine autoimmune models used to evaluate therapeutic effects of Gal-9 rely on specific antibodies or disease inducing peptides in combination with infection stimulating adjuvants and/or bacteria [3], [19], [9]. In contrast, the CTL stimulatory effects via dendritic cell (DC) activation and induction of IFN-γ found in a sarcoma did not require additional skewing stimuli [12]. Together, this suggests that the outcome of Gal-9 signaling varies greatly, depending on experimental conditions and/or the balance of immunity in specific disease settings.

Here, we aimed to establish the effect of Gal-9 treatment on freshly isolated and non-skewed human peripheral blood immune cells in the absence of other stimuli. In line with current thinking, Gal-9 triggered cell death in >95% of T-cells at high concentrations. However, at lower doses, Gal-9 activated and strongly expanded surviving T-cells in a TIM-3-independent manner. In addition, the T-cell expansion induced by Gal-9 was characterized by a shift from a naïve towards a central memory (T_cm_) and IFN-γ producing T_H1_ phenotype. Further, a shift in monocytes towards a DC-phenotype was detected. However, monocyte depletion did not affect T-cell activation, indicating that Gal-9 had a direct effect on T-cells. In the presence of anti-CD3/IL-2 T-cell activation signals, Gal-9 did not trigger expansion of T-cells, but shifted the normal CD8/CD4 balance towards a predominant CD4^+^ phenotype. Taken together, these data indicate that Gal-9 has diverse immunomodulatory effects depending on concentration and skewing signals available and is therefore likely to have a disease-specific role that needs to be evaluated in depth for both autoimmunity and cancer.

## Materials and Methods

### Antibodies & reagents

The following fluorophore-conjugated anti-human antibodies from Immunotools (Friesoythe) were used in this study; anti-CD3-FITC (MEM-57), anti-CD3-Dy647 (MEM-57), anti-CD4-PE (MEM-241), anti-CD4-PE/Dy647 (MEM-241), anti-CD8-Pe/Dy47 (MEM-31), anti-CD14-Dy647 (MEM-15), anti-CD19-APC (LT19), anti-CD25-PE (MEM-181), anti-CD62L-FITC (LT-TD180). The following anti-human antibodies were from eBioscience; anti-CD8-PEcy7 (RPA-T8), anti-CD45RO-APC (UCHL1), anti-CD56-APC (MEM188), anti-TIM-3-APC (F38-2E2), anti-PD-1-PERCP (eBioI105), anti-CCR7-PerCP-Cy5.5 (3D12), anti-FoxP3-APC (236A/E7), anti-IL-2-PERCP-Cy5.5 (MQ1-17H12), anti-IL-4-PE-Cy7 (8D4-8), anti-IFNy-PERCP-Cy5.5 (4S-B3), and anti-IL-17-PERCP-Cy5.5 (eBio64DEC17). The anti-CD3-CyQ antibody was from IQ-products (IQP-519C). Streptavidin-Alexa488 was from Invitrogen. Recombinant Gal-9 (truncated form Gal-9(0)) and the physiologically occurring short isoform of Gal-9 (Gal-9(S)) were produced as described before [20]. Gal-1, Gal-2, Gal-3, Gal-8 were purchased commercially (R&D systems).

### Isolation of peripheral blood mononuclear cells (PBMCs) and activation of T-cells

Peripheral blood mononuclear cells (PBMCs) were obtained from venous blood from healthy volunteers using standard density gradient centrifugation (Lymphoprep). The ethics review board of the Multi- Regional Ethics Committee approved the study (MREC 06/Q2102/56), and all blood samples were obtained with written consent from the healthy subjects. Activated T-cells were generated by culturing PBMCs with anti-CD3 mAb (0.5 µg/mL; 72 h, UCHT1, Immunotools) followed by 96 h IL-2 (100 ng/mL, Immunotools). Monocyte depletion prior to treatment was performed by magnetic-activated cell sorting (MACS) using anti-CD14-beads (Miltenyi Biotec), resulting in >99% depletion of monocytes as verified by flow cytometry (data not shown). Cells were cultured at 37°C exposed to 21% O_2_/5% CO_2_ in X-VIVO medium (Lonza), a chemically defined and serum-free hematopoietic cell medium, or RPMI-1640 (Lonza) supplemented with 10% v/v FBS (Thermo Scientific). Cell numbers were quantified using a cell counter (Sysmex).

### Flow cytometry

All flow cytometric analyses were performed on a BD Accuri C6 flow cytometer (BD Biosciences) and accessory CFlow Plus analysis software. Positively and negatively stained populations were calculated by quadrant dot plot analysis (for representative dot-plots of stainings see Figures S1–S3). For cell surface marker analysis with antibodies, viable cells were gated based on forward and sideward scatter plot (for example see Figure S1B).

### Assessment of apoptosis and Gal-9 binding

PBMCs were treated for 1–7 days with medium with or without the indicated concentration of Gal-9 and analyzed for apoptosis using flow cytometric staining for phosphatidylserine exposure using an Annexin-V-FITC staining kit according to the manufacturer’s protocol (Immunotools). In brief, cells were washed once with calcium binding buffer, resuspended in calcium buffer with Annexin-V, incubated for 10 min at 4°C, and analyzed by flow cytometry. To determine if Gal-9 bound to PBMCs in a CRD-dependent manner, competitive binding experiments were performed in which cells were incubated with biotinylated Gal-9 for 1 h, in the presence and absence of 40 mM α-lactose, followed by 3 washes to remove excess and non-bound Gal-9. Cell surface binding was detected using streptavidin-Alexa488.

### Phenotypic analyses

Phenotype assessment was determined by immunofluorescent flow cytometric staining essentially as described before [21]. Briefly, distribution of cell populations was analyzed gating on peripheral blood lymphocytes from forward scatter/sideward scatter, detecting CD3^+^ and CD19^+^ populations. In addition, CD3^−^/CD19^−^ cells were gated and analyzed for CD56-PE binding. The balance of CD4 and CD8 was analyzed within the viable cell population gated from forward scatter/sideward scatter using anti-CD4-PE and anti-CD8-PEcy7. T_EM_ staining was performed on 0.5×10^6^ cells per condition by incubation of PBMCs with APC-conjugated anti-CD45RO, PERCP-Cy5-conjugated anti-CCR7, and FITC-conjugated anti-CD3 for 30 min at room temperature in the dark. Alternatively, cells were stained with FITC-conjugated anti-CD62L, CyQ-conjugated anti-CD3 and APC-conjugated CD45RO. Isotype-matched non-specific antibodies were used as negative controls. Staining was analyzed on viable cells on forward scatter/sideward scatter plot selected within the CD3^+^ cell population.

### Carboxyfluorescein succinimidyl ester (CFSE) analysis

To determine the number of cell divisions after Gal-9 treatment, a CFSE cell proliferation kit was used according to manufacturer’s protocol (CellTrace™ CFSE Cell Proliferation Kit, Invitrogen). In brief, harvested PBMCs were stained with 2.5 µM CFSE in 0.1% w/v BSA/PBS for 10 min at 37°C. Subsequently, PBMCs were incubated in fresh medium for 10 min on ice and excess dye was removed by 3 washes. CFSE-stained cells were then used to establish *in vitro* cell cultures. During a time course of 7 days, cells were harvested and an additional staining was performed, incubating CFSE-labeled PBMCs with anti-CD3-Dye647, anti-CD4-PE/Dye647, anti-CD8-PE/Dye647, anti-CD19-APC or CD56-APC for 30 min at 4°C. Staining was analyzed gating on the cell populations positive for the different phenotypic cellular markers, in which their CFSE-staining pattern was detected.

### T_H1_, T_H2_, T_H17_ and regulatory T-cell (T_reg_) immunofluorescence staining

To determine the effect of Gal-9 treatment on T_H1_, T_H2_, T_H17_, PBMCs were treated for 7 days with medium supplemented with or without 15 nM Gal-9, then washed and subsequently stimulated with phorbol 12-myristate 13-acetate (PMA) for 4 h. Subsequently, cells were washed in wash buffer (PBS, 5% v/v FBS, 0.1% w/v sodium azide) and stained with FITC-conjugated anti-CD3 for 15 min at room temperature. Cells were fixed with Reagent A (Caltag) for 10 min. After washing, cells were resuspended in permeabilization Reagent B (Caltag) and labeled with PERCP-Cy5-conjugated anti-IL-2, PERCP-Cy5-conjugated anti-IL-4, PERCP-Cy5-conjugated anti-IFN-γ, or PERCP-Cy5-conjugated anti-IL-17 for 20 min in the dark. Cells were analyzed for T-cell phenotype after 2 subsequent washes with PBS. Cytokine staining was performed on CD3^+^-gated cells. T_reg_ staining was performed by cell surface staining of PBMCs with PE-conjugated anti-CD4, FITC-conjugated anti-CD3, fixation/permeabilization as for cytokine staining above, and subsequent intracellular staining for the transcription factor FoxP3 with APC-conjugated anti-FoxP3. Staining for FoxP3 was assessed within the CD3 and CD4 double-positive population of cells.

### Analysis of cytokine secretion

ELISAs were used to quantify the secretion of IFN-γ and IL-17 from PBMCs treated with and without Gal-9. In brief, *in vitro* experiments were set-up as described above and supernatant of treated cells was collected at day 7. The IFN-γ (Immunotools) and IL-17 (Thermo Scientific) ELISAs were performed according to manufacturer’s protocol.

### Statistical analysis

Statistical analysis was performed by one-way ANOVA followed by Tukey-Kramer post-test or, where appropriate, by two-sided unpaired Student’s t-test using Prism software. p<0.05 was defined as a statistically significant difference. Where indicated *  = p<0.05; **  = p<0.01; ***  = p<0.001.

## Results

### Galectin-9 triggers cell death but also expands resting peripheral blood cells

**Figure 1:**
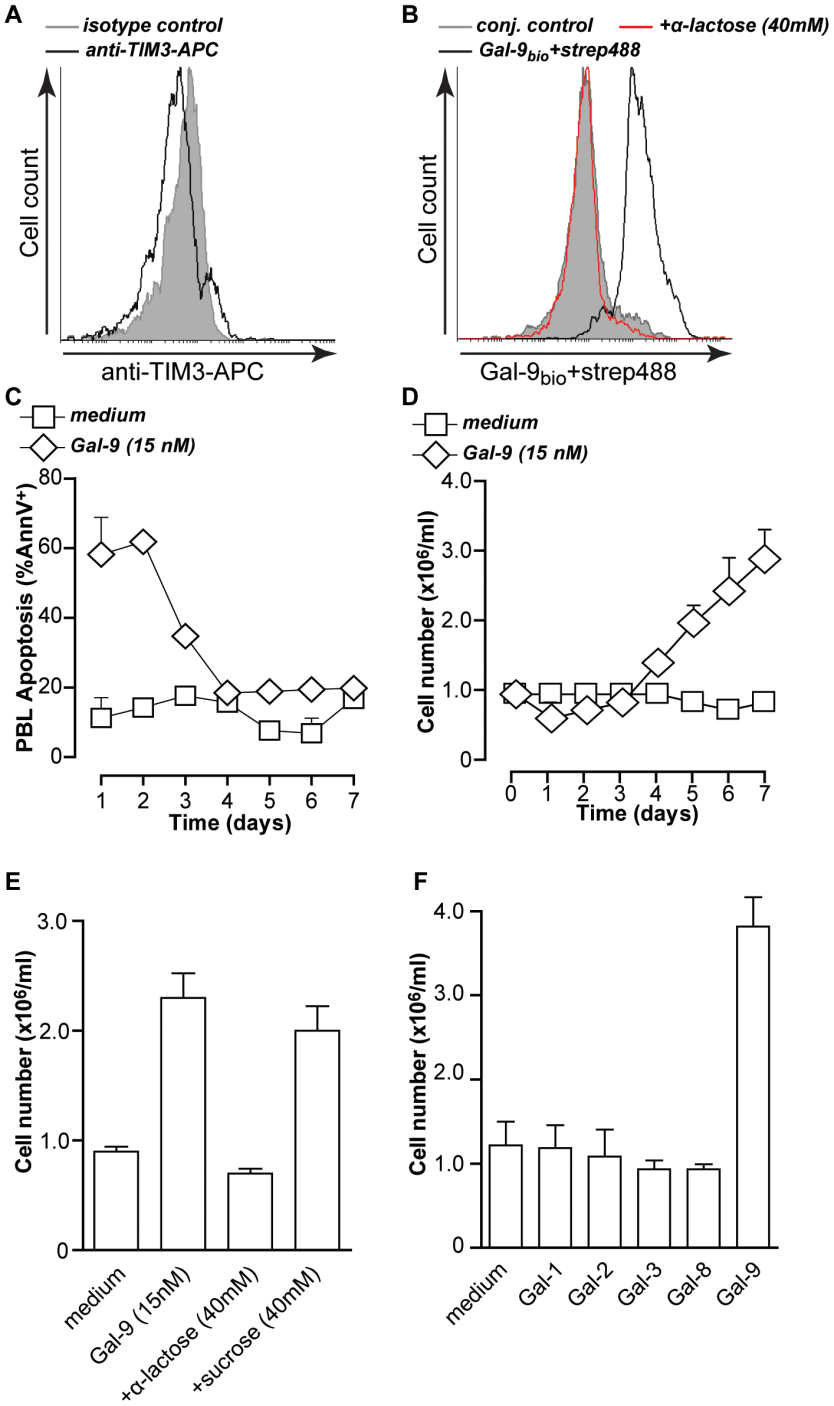
Gal-9 triggers TIM-3-independent cell death and PBMC expansion. **A.** Resting PBMCs were incubated with anti-TIM3-APC or isotype control antibody or **B.** with streptavidin-Alexa488, biotinylated Gal-9+streptavidin-Alexa488 in the presence or absence of α-lactose. Cell surface staining was evaluated by flow cytometry **C.** Resting PBMCs (n = 3) were treated with medium or 15 nM of recombinant Gal-9 for up to 7 days. Cell death was determined by Annexin-V staining. **D.** PBMCs (n = 8) treated as in (C) were analyzed for cell number. **E.** Resting PBMCs (n = 8) were treated as in (C) in the presence of α-lactose or sucrose, and analyzed for cell number. **F.** Resting PBMCs (n = 3) were treated with medium or 15 nM of recombinant Gal-9, Gal-1, Gal-2, Gal-3, or Gal-8 for up to 7 days, after which cell number was determined. All graphs represent mean +/− SD.

Initial evidence suggested that the predominant effect of treatment of T-cells with Gal-9 is the induction of apoptotic cell death in T-helper 1 (T_H1_) and T-helper 17 (T_H17_) cells via the receptor TIM-3 [3]. However, flow cytometric analysis on resting PBMCs revealed that expression of TIM-3 was negligible, whereas *ex vivo* activated T-cells did express TIM-3 (Figure 1A and Figure S1A). Despite the lack of cell surface-expressed TIM-3 Gal-9 bound to resting PBMCs (Figure 1B). Binding of Gal-9 was inhibited in a lectin-specific manner by co-incubation with 40 mM α-lactose (Figure 1B), but not by an anti-TIM-3 blocking antibody (data not shown). Thus, the binding of Gal-9 was carbohydrate recognition domain (CRD)-dependent and TIM-3-independent.

Treatment of PBMCs with Gal-9 at a concentration of 150 nM, falling within the previously published range, eliminated the vast majority of cells within 1 day (Figure S1B). However, a 10-fold lower concentration of Gal-9 (15 nM) only triggered cell death in ∼half of the cells (Figure S1B). Quantification of cell death during prolonged treatment of up to 7 days, revealed that the percentage of apoptotic cells in Gal-9 treated conditions gradually declined from ∼60% at day 1 to that of medium control within 4 days (Figure 1C). In line with this, treatment with 15 nM Gal-9 decreased cell counts compared to control at days 1–3 (Figure 1D). This initial depletion was followed by strong PBMC expansion, as evidenced by a 3-fold increase in cell count at day 7 from 1×10^6^ cells/ml to ∼3×10^6^ cells/ml in Gal-9 treated conditions (Figure 1D). This PBMC expansion by Gal-9 was inhibited by co-treatment with α-lactose, but not sucrose, and thus CRD-dependent (Figure 1E).

Various other immunomodulatory members of the galectin family, i.e. Galectin-8, which is a tandem-repeat Galectin like Gal-9, as well as Galectin-1, Galectin-2 (prototypic Galectins) and Galectin-3 (chimeric Galectin) did not trigger significant increase of PBMC counts at these concentrations (Figure 1F), nor significant induction of apoptosis (data not shown). Thus, in addition to initial induction of apoptosis, Gal-9 triggers expansion of peripheral blood cells.

### Galectin-9 and the short isoform of Gal-9 (Gal-9(S)) dose-dependently activate T-cells

**Figure 2:**
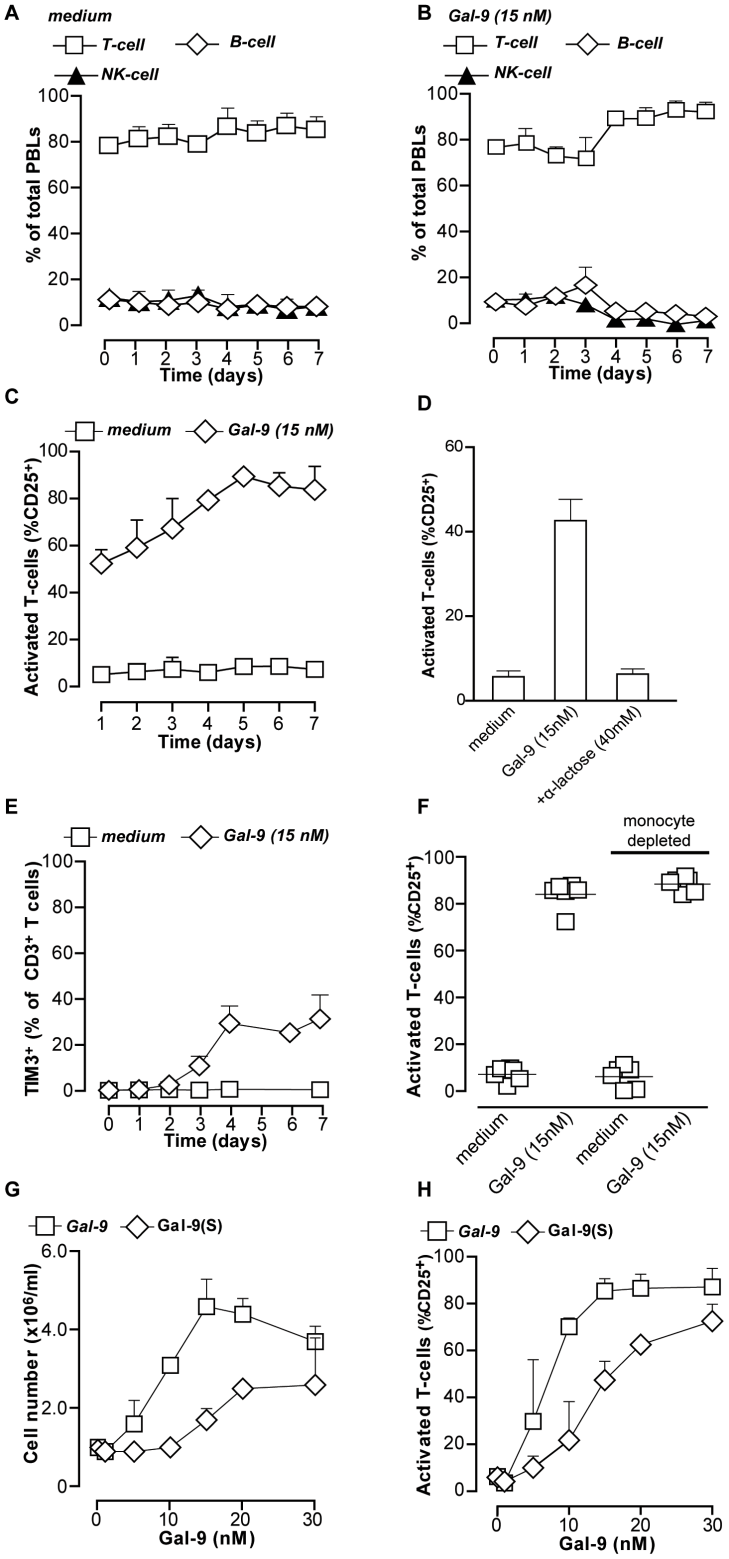
Recombinant and Gal-9(s) dose dependently activate T-cells. **A.** Resting PBMCs (n = 5) were incubated in medium for up to 7 days. Distribution of cell populations was analyzed every day by flow cytometry. **B.** Similar as (A), but in the presence of 15 nM Gal-9. **C.** Resting PBMCs (n = 5) treated with medium or 15 nM of recombinant Gal-9 for up to 7 days were analyzed for T-cell activation by staining for activation marker CD25. **D.** Resting PBMCs were treated as in (C) in the presence of α-lactose, after which CD25 expression was analyzed at 1 day. **E.** Resting PBMCs (n = 5) were treated as in (C) and analyzed for expression of TIM-3. **F.** PBMCs or monocyte-depleted PBMCs were treated with Gal-9 for 7 days, and analyzed for CD25 expression **G.** Resting PBMCs (n = 6) were treated with a concentration range of recombinant Gal-9 or physiologically occurring isoform Gal-9(S) for 7 days and analyzed for cell density. **H.** Resting PBMCs (n = 6) were treated as in G and CD25 expression was determined. All graphs represent mean +/– SD.

To specify the stimulatory effect of Gal-9 on the PBMC fraction, the relative proportion of T-cells, B-cells and NK-cells was analyzed in medium and Gal-9 treated PBMCs. In medium control, the percentages of all lymphocyte populations remained stable from day 0–7 (Figure 2A). In contrast, treatment with Gal-9 reduced the percentage of CD3^+^ positive T-cells during the first 3 days, followed by a concomitant increase in T-cells and decrease in B-cells and NK-cells from day 4 onwards (Figure 2B). Further, in the viable population of CD3^+^ T-cells, treatment with 15 nM Gal-9 also triggered T-cell activation, as evidenced by the upregulation of surface CD25-expression from ∼50% at day 1 to a maximum of ∼80% after 5–7 days (Figure 2C, representative dot-plots in Figure S1C). Activation of T-cells by Gal-9 was inhibited by co-treatment with α-lactose and thus CRD-dependent (Figure 2D). Other galectin family members did not trigger T-cell activation (Figure S1D). Interestingly, during this time-course, the expression of TIM-3 was induced in only 20–30% of T-cells after 4–7 days of treatment with Gal-9 (Figure 2E). These T-cells did not become double-positive for TIM-3 and PD-1 (Figure S2E), a marker profile associated with T-cell exhaustion [22]. Thus, Gal-9 interacted with T-cells via an as yet unidentified receptor, resulting in apoptosis of part of the T-cells. However, the remaining population of viable T-cells underwent activation as well as strong expansion.

In addition to lymphocytes, the isolated PBMC fraction contains monocytes. These monocytes responded to Gal-9 treatment with a marked stretching and loss of CD14 expression, indicative of a shift towards a DC-phenotype (Figure S2A). Importantly, depletion of monocytes from the PBMC population prior to Gal-9 treatment did not affect Gal-9-induced activation of T-cells, as determined by CD3/CD25 double staining (Figure 2F). Thus, although Gal-9 activated monocytes, this was not required for activation and expansion of T-cells.

In addition to recombinant Gal-9, the short isoform of Gal-9 (Gal-9(S)) was included to evaluate whether naturally occurring isoforms of Gal-9 could have similar immunomodulatory effects. Both Gal-9 and Gal-9(S) dose-dependently triggered activation and expansion of T-cells, with maximal effects of recombinant Gal-9 at 15 nM (0.5 µg/ml) and Gal-9(S) at 30 nM (1 µg/ml) (Figure 2G and 2H). Thus, both recombinant Gal-9 and Gal-9(S) triggered dose-dependent activation of T-cells at low concentrations.

### Galectin-9 treatment results in expansion of CD4^+^ T-cells

**Figure 3:**
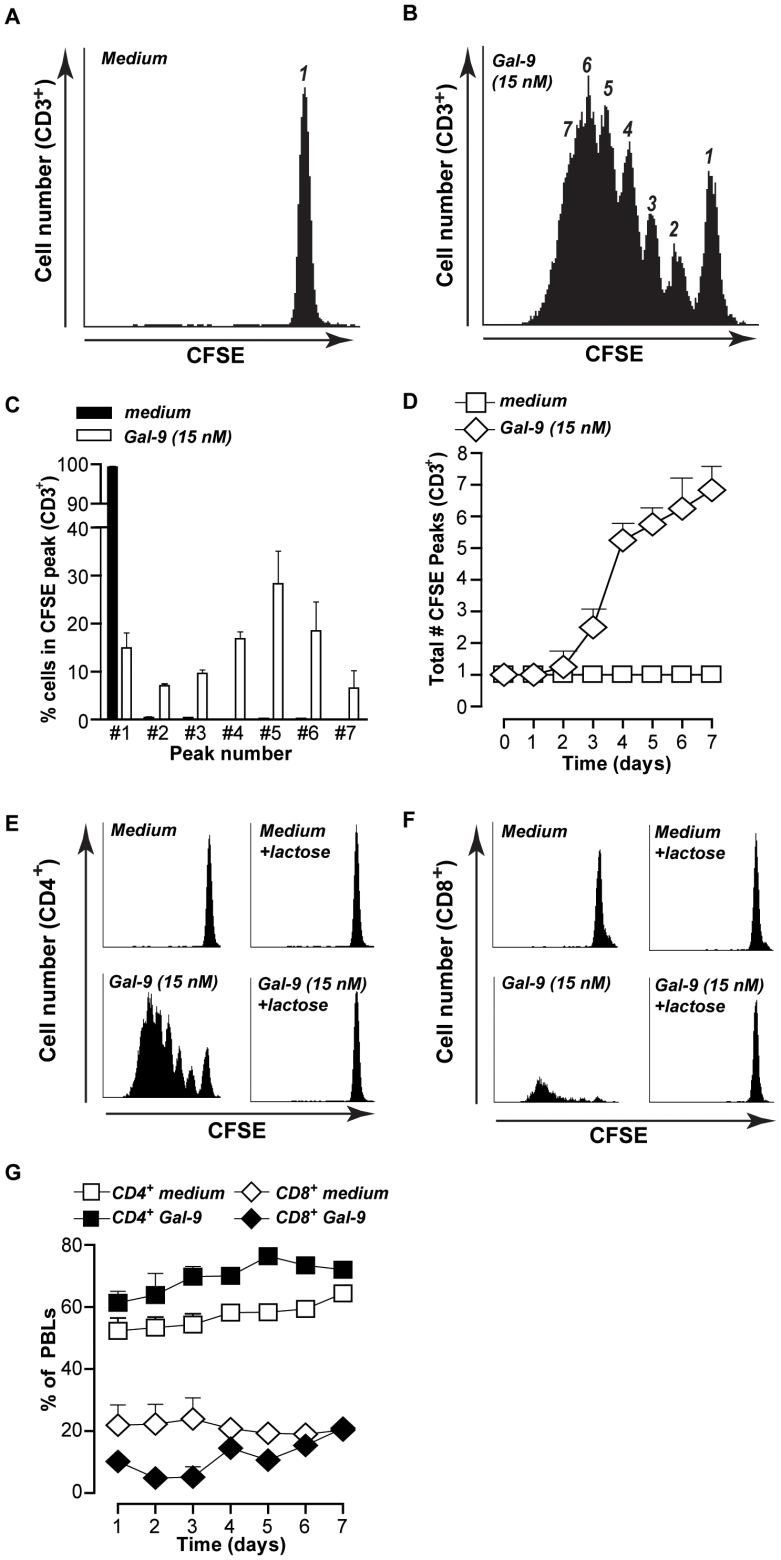
Galectin-9 treatment expands CD4^+^ T-cells. **A.** Representative plot of 6 independent experiments of resting PBMCs stained with CFSE and subsequently incubated in medium for up to 7 days. At Day 7, PBMCs were harvested, stained with the T-cell marker CD3, and CFSE peak pattern was analyzed within the CD3^+^ cells by flow cytometry. **B.** Representative plot of 6 independent experiments of resting PBMCs as treated in A, but in the presence of 15 nM Gal-9. **C.** Analysis of (A) and (B) showing percentage of CD3^+^ T-cells in the respective peak of all independent experiments (mean +/− SEM). **D.** Analysis of (B) showing the number of CFSE peaks of all independent experiments. **E.** Representative plots of 3 independent experiments of resting PBMCs stained with CFSE and subsequently incubated in medium or 15 nM Gal-9 (+/− lactose) for up to 7 days. At Day 7, PBMCs were harvested, stained with the T-cell marker CD4, and CFSE peak pattern was analyzed within the CD-3^+^ cells by flow cytometry. **F.** As in (E) but stained for the T-cell maker CD8. **G.** Resting PBMCs (n = 4) were treated for up to 7 days with medium or Gal-9 and analyzed for CD4 and CD8 distribution. All graphs represent mean +/− SD unless stated otherwise.

The effect of treatment of T-cells with Gal-9 on cell division was evaluated by determining T-cell proliferation by CFSE staining. In control conditions, the CD3^+^ T-cells did not divide during the 7 day time-course, as evidenced by a single CFSE fluorescence peak (Figure 3A). In contrast, treatment with Gal-9 triggered up to 6 cell divisions within the CD3^+^ T-cell population (Figure 3B), with ∼7% of T-cells having divided 6 times at day 7 (Figure 3C). In line with the data on apoptosis and cell counts, T-cell division was first detected after 3 days of Gal-9 treatment (Figure 3D).

Further analysis within the Gal-9 treated populations identified that most dividing T-cells were CD4^+^ (∼76% of total PBMCs at day 7, Figure 3E), although the remaining CD8^+^ T-cells (∼20%) also divided in the final days (Figure 3F). In line with this, Gal-9 treatment induced a steady increase in the percentage of CD4^+^ T-cells during the 7 day time-course, whereas the percentage of CD8^+^ T-cells initially decreased, but returned to base levels at day 7 (Figure 3G; representative dot-plot in Figure S2B). Again, Gal-9 induced effects were CRD-specific as α-lactose completely inhibited cell division in both CD4^+^ and CD8^+^ T-cells (Figure 3E and 3F).

### Galectin-9 expands central memory and T-helper 1 cells

**Figure 4:**
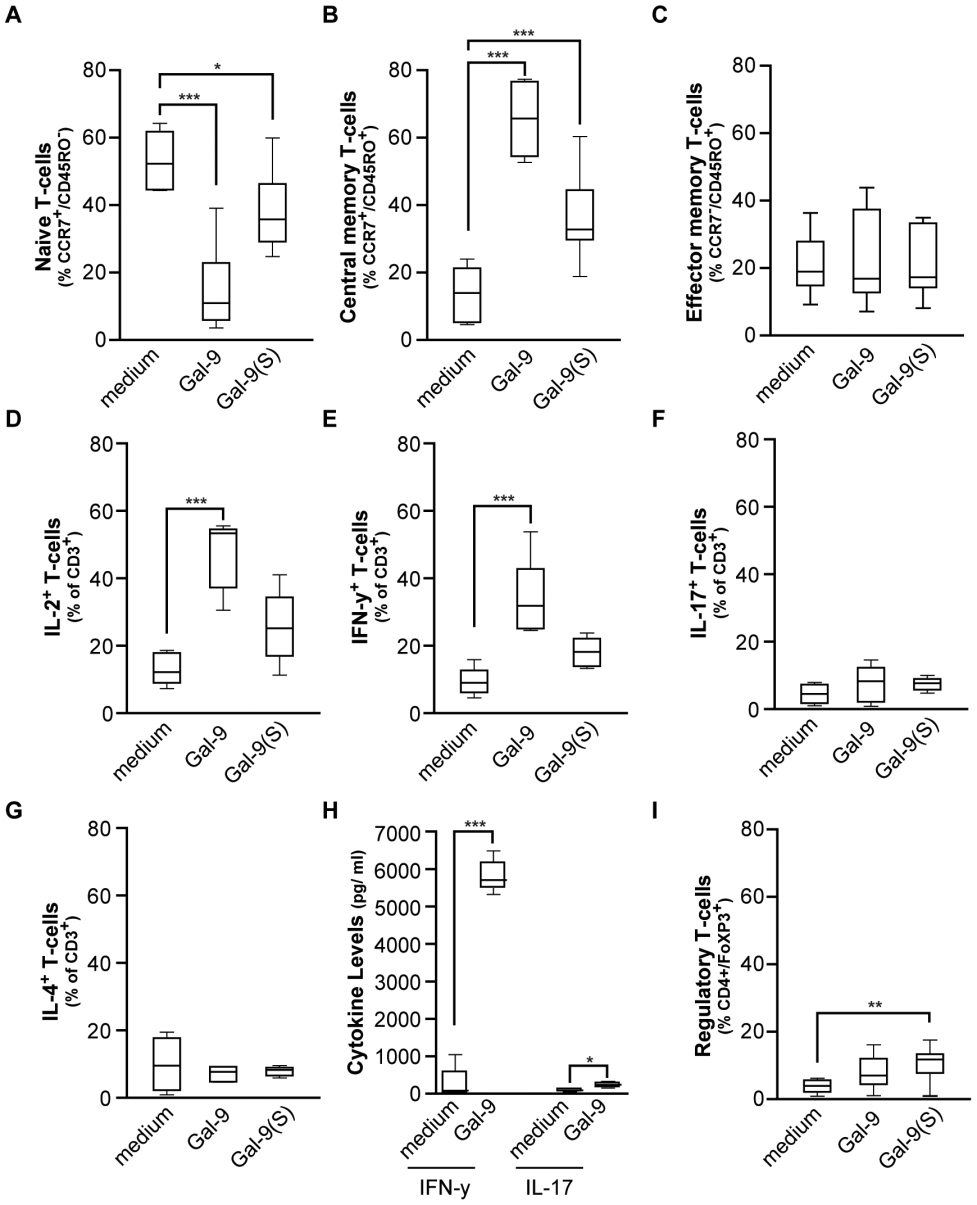
Gal-9 treatment of resting PBMCs shifts T-cells towards central memory and T-helper 1 phenotype. **A**−**C.** PBMCs were treated for 7 days with Gal-9 or Gal-9(S) after which the percentage of naïve (**A**), central memory (**B**), and effector memory (**C**) was evaluated by flow cytometry. **D**−**G**. Resting PBMCs were treated for 7 days with Gal-9 or Gal-9(S), after which T-cell cytokine production was analyzed by flow cytometry as described in M&M. The percentage of IL-2 (n = 11) (**D**), IFN-γ (**E**), IL-17 (**F**) and IL-4 (**G**) was determined. **H.** Supernatants of medium and Gal-9 (15 nM) treated PBMCs was harvested at day7 after which the amount of secreted IFN-γ and IL-17 was determined with ELISA. **I.** PBMCs (n = 11) were treated for 7 days with Gal-9 or Gal-9(S) after which the percentage of regulatory T-cells was determined. Unless indicated otherwise; (n = 12).

As Gal-9 clearly activated and expanded T-cells, the relative proportion of naïve, central memory (T_CM_), and effector memory (T_EM_) phenotypes in the PBMCs was evaluated (for representative dot-plots of staining see Figure S2C). As expected, the majority of T-cells in medium control were of a naïve CCR7^+^/CD45RO^−^ phenotype (Figure 4A). However, upon treatment with Gal-9 or Gal-9(S), the percentage of naïve T-cells was strongly and statistically significantly reduced (Figure 4A), with the majority of T-cells acquiring a central CCR7^+^/CD45RO^+^ memory phenotype (Figure 4B). This dramatic shift was confirmed by a second staining for central memory using CD62L and CD45RO, which revealed a similar increase in CD62L^+^/CD45RO^+^ T_CM_ (Figure S2D). In contrast, no significant changes were detected in the CCR7^−^/CD45RO^+^ T_EM_ population (Figure 4C).

To characterize T-cells expanded by Gal-9 on a more functional level, cytokine profiles were determined in medium control and Gal-9 treated T-cells (see Figure S3 for representative dot-plots). In line with the immunophenotyping data, there was a marked and statistically significant increase in IL-2 producing T-cells, indicative of T_CM_ cells (Figure 4D). In addition, treatment with Gal-9 resulted in a statistically significant increase in IFN-γ producing T-cells (Figure 4E), but not in IL-17 and IL-4 producing T-cells (Figure 4F and 4G, respectively). In line with these findings, analysis of secreted cytokines within the supernatant of Gal-9 treated cells revealed an ∼100-fold increase in IFN-γ levels compared to medium control at day 7 (Figure 4H). Further, a low but significant increase in IL-17 was also detected upon treatment with Gal-9 (Figure 4H). In line with earlier findings, treatment with 15 nM Gal-9(S) but not Gal-9 also triggered a small but statistically significant increase in regulatory T-cells (T_regs_) after 7 days (Figure 4I). Thus, in resting PBMCs the treatment with Gal-9 triggers a selective expansion of central memory T-cells and a predominant induction of IFN-γ-producing CD4^+^ T-cells.

### T-cell receptor-mediated activation in the presence of Gal-9 shifts the balance towards CD4+ T-cells

**Figure 5:**
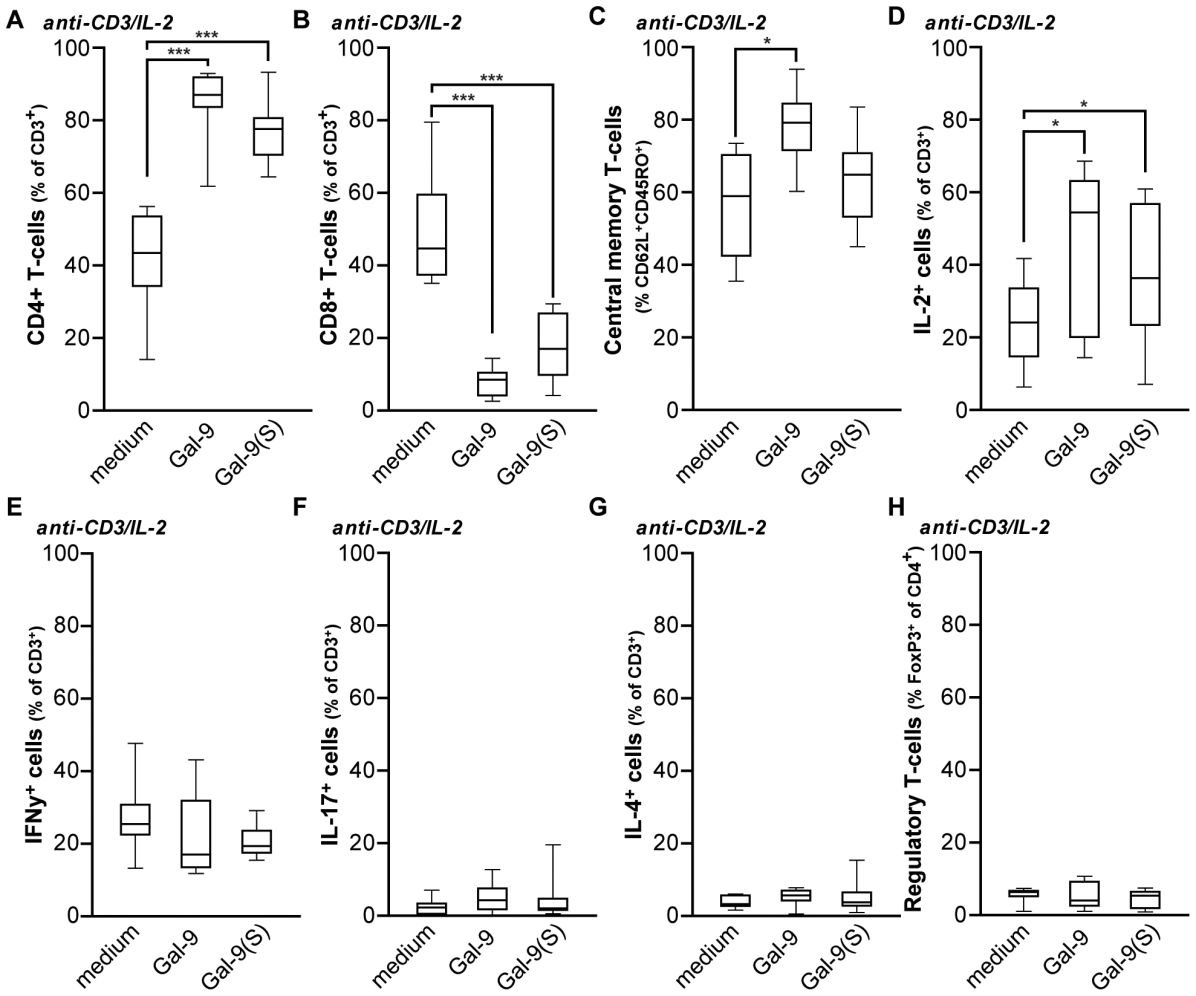
Gal-9 treatment of TCR-activated T-cells reverses CD8/CD4 distribution and shifts T-cells towards a central memory phenotype. A−B. T-cells were activated by anti-CD3/IL-2, or additionally with 15 nM Gal-9 or Gal-9(S). After 7 days the percentage of CD4 (A) and CD8 (B) were determined by flow cytometry. C. T-cells were activated as in (A) and percentage of T-cells with central memory phenotype was determined. D−G. T-cells were activated as in A, after which T-cell cytokine production was analyzed by flow cytometry as described in M&M. The percentage of IL-2 (n = 11) (D), IFN-γ (E), IL-17 (F) and IL-4 (G) was determined. H. T-cells were activated as in (A), after which the percentage of regulatory T-cells was determined. Unless indicated otherwise (n = 12).

The results above provide evidence that Gal-9 has a potent immunomodulatory effect on resting T-cells. This T_H1_-stimulatory effect is in line with a recent report in which CD3/CD28 co-stimulation of T-cells stimulated T_H1_ development [13]. To further characterize the modulatory effect of Gal-9 during T-cell receptor (TCR)-stimulation, PBMCs were stimulated with anti-CD3 antibody for 3 days, followed by IL-2 stimulation for 4 days. In the absence of Gal-9, this stimulation regime led to a slight preferential induction of CD8^+^ T-cells compared to CD4^+^ T-cells (Figure 5A and 5B; median CD4^+^ 42.7% vs. median CD8^+^ 47%). However, treatment with Gal-9 or Gal-9(S) induced a dramatic shift in percentages of CD8^+^ and CD4^+^ T-cells after 7 days of activation (Figure 5A and 5B; median CD4^+^ 86.1% vs. median CD8^+^ 7.2%). Of note, no significant increase in apoptotic cells or cell counts was detected upon treatment with Gal-9 or Gal-9(S) after 7 days of treatment (data not shown). TCR/IL-2 mediated activation of T-cells in the presence of Gal-9 shifted T-cells toward a central memory phenotype, as defined by immunophenotyping for CD45RO/CCR7 or CD45RO/CD62L (Figure 5C), and intracellular cytokine staining for IL-2 (Figure 5D). In contrast to treatment of resting PBMCs, treatment of anti-CD3/IL-2 activated T-cells with Gal-9 did not trigger an increase in the percentage of IFN-γ producing cells (Figure 5E). Similarly, no increase in IL-17 or IL-4 producing T-cells or regulatory T-cells was observed (Figure 5F−H). Taken together, TCR-mediated activation and IL-2 induced expansion of T-cells in the presence of Gal-9 shifts the T-cell response towards a CD4^+^ helper response that is further characterized by a central memory phenotype.

## Discussion

Gal-9 is a strong modulator of T-cell immunity known for its apoptotic effects on T_H1_ and T_H17_ cells in autoimmunity, but also for its stimulatory activity on CTLs and T_H1_ cells in cancer and food allergy. These apparent diametrical outcomes of Gal-9 signaling on T-cell immunity suggests that the balance of immunomodulatory signals is crucial for Gal-9 signaling. Data presented in the current study indicate that single-agent treatment of resting PBMCs with low Gal-9 doses is, after initial apoptotic elimination, accompanied by activation and subsequent expansion of CD4^+^ T-cells. Furthermore, Gal-9 treatment shifts T-cells from a naïve to a central memory phenotype and increases the percentage of IFN-γ producing T-cells. In activated (anti-CD3/IL-2) T-cells, Gal-9 skews the CD4^+^/CD8^+^ balance towards a CD4^+^ phenotype.

As evident from the data, the reported differences in T-cell responses upon Gal-9 treatment can be partly ascribed to the amount of Gal-9 that is used. In particular, a concentration of 150 nM eliminated the vast majority of T-cells within one day, whereas a dose-response analysis demonstrated that 15–30 nM Gal-9 is the optimal concentration for T-cell stimulatory effects. In previous reports, T-cell apoptosis was induced with relatively high doses of up to 1000 nM Gal-9 [23], [3]. Furthermore, a recent study using murine T-cell clones also required high Gal-9 concentrations to induce T-cell apoptosis (400 nM), whereas non-lethal doses had T-cell stimulatory effects [15]. In addition to concentration, most studies were designed to evaluate Gal-9 activity at relatively short incubation periods varying from 1h to 4 days [3], [7], [4], [23], [24]. In the current study, Gal-9 effects were evaluated for up to 7 days. Notably, significant T-cell expansion in the current study was only seen after 4 days of treatment, whereas the induction of apoptosis occurred rapidly within 1 day. Of note, these effects were independent of TIM-3, as freshly isolated T-cells lacked TIM-3 expression, and only ∼35% of T-cells became TIM-3^+^ after 4-7 days of Gal-9 treatment. Such TIM-3 independent binding has been described earlier [15], [6], [3], [7], and several alternate binding partners have been reported, e.g. CD40 [18], several adhesion molecules [25], [26], immunoglobulin E (IgE) [27], and protein disulfide isomerase [16], [17]. Whether the TIM-3 independent binding observed in the current study can be attributed to binding of Gal-9 to one of these alternate receptors, or an as yet unidentified receptor, is subject of an ongoing study.

Binding of Gal-9 to resting blood lymphocytes activated T-cells and shifted T-cell phenotype from naïve toward IL-2 producing central memory T-cells (CD62^+^/CCR7^+^/CD45R0^+^) and IFN-γ producing T_H1_ cells. This single-agent stimulatory effect of Gal-9 on resting T-cells has not been reported before and appears to contrast with published studies that describe predominant elimination of T_H1_ cells by Gal-9. However, our findings are in line with several recent reports on the stimulatory effect of Gal-9 on activated T_H1_ cells [13], [15], and the induction of central memory cytokines by Gal-9 upon CD3/CD40 co-stimulation in murine T-cells [18]. The mechanism by which Gal-9 induces T-cell activation and proliferation is currently unknown. However, other lectins, such as concanavalin A, also potently stimulate T-cell proliferation. Concanavalin A does so by directly interacting with activating receptors, like CD3 [28]. Within the Galectin family of lectins, it has been reported that Galectin-1 can directly bind to CD3 on T-cells [29]. It was suggested that Galectin-1 mediated ligation of the CD3-complex mimics antigen-induced TCR signaling, which induces early events in T-cell activation comparable to those elicited by agonistic anti-CD3 antibodies. Therefore, it is tentative to speculate that Gal-9 interacts with activating receptors such as CD3 on the T-cell surface. This is currently subject for further study in our laboratory.

Besides direct Gal-9 effects on T-cells, indirect T-cell stimulation by Gal-9 via the activation of DCs and DC-like macrophages was reported in sarcoma and melanoma bearing mouse models [12], [25]. Furthermore, Gal-9 induced the maturation of human monocyte-derived dendritic cells, resulting in IL-12, IL-2 and IFN-γ secretion [30]. In line with these findings, monocytes did undergo phenotypic changes towards DC-phenotype in the current study. However, monocyte depletion did not affect the activity of Gal-9 towards resting T-cells, which suggests that Gal-9 has a direct immunostimulatory effect on T-cells. Gal-9 was also reported to enhance production of IFN-γ by NK-cells [31], but our analysis of lymphocyte distribution/activation in the current study showed only an increase in T-cells and a decrease in NK-cells. Further, a clear increase in IFN-γ producing T-cells was detected. Thus, our data suggest that IFN-γ is secreted by T-cells and not NK-cells. In addition to T_H1_ cells, a statistically significant increase in T_reg_ cells by Gal-9(S) was detected, which is in line with recent murine studies [32], [33].

The here reported activating effect of low dose Gal-9 on resting T-cells may be of relevance in certain human diseases. For instance, elevated Gal-9 serum levels were detected in patients with type 2 diabetes and chronic kidney disease [34]. Here, serum Gal-9 levels negatively correlated with renal function and increased along with disease progression. Of note, aberrant recruitment and activation of T-cells has been described in diabetic nephropathy [35]. Hence, Gal-9 may be involved in T-cell activation, which contributes to kidney damage in diabetes type 2. In addition, dietary supplementation of pre-biotic oligosaccharides and *Bifidobacterium breve* reduced allergic symptoms in a murine model of food allergy and in infants with atopic dermatitis, as an effect of increased serum Gal-9 levels and subsequent T_H1_ and T_reg_ responses [13]. Thus, depending on type of disease and immunological environment, serum Gal-9 can have both positive and negative effects on disease progression by the activation of T-cells.

In conclusion, treatment of human resting blood T-cells with Gal-9 induces apoptosis in a substantial proportion of the cells, but also activates and expands IFN-γ producing T_H1_ cells and central memory T-cells in surviving population. In the presence of activating signals (anti-CD3/IL2), the treatment with Gal-9 does not expand T-cells, but skews the CD4^+^/CD8^+^ balance towards a CD4^+^ phenotype. This study thus uncovers the stimulatory effect of Gal-9 treatment on resting lymphocytes and highlights the complexity of immunomodulatory signaling by Gal-9 on human T-cells. Indeed, in various diseases settings the influence of Gal-9 on T-cell immunity will be determined by micro-environmental concentrations of Gal-9 and other immune modulators as well as the activation status of T-cells.

## Supporting Information

Figure S1 **A.** T-cells were activated with anti-CD3 (72h) and IL-2 (96h), after which cell surface expression of TIM-3 was analyzed by flow cytometry. **B.** resting PBMCs were treated with medium, 15 nM or 150 nM of Gal-9 for 1 day. Representative fsc/ssc dot-plots of lymphocytes demonstrate that treatment with 150 nM of Gal-9 shifts cells from a viable population (see medium; left panel) to dead/fragmented distribution (150 nM Gal-9; right panel), whereas at 15 nM ∼50% of cells remain viable (15 nM Gal-9; middle panel). **C.** representative flow cytometric dot-plots of CD3/CD25 staining **D.** resting PBMCs were treated with 0-15 nM Gal-1, Gal-2, Gal-3, Gal-8 or Gal-9. **E.** representative flow cytometric dot-plots of TIM-3/PD-1 staining in which cells were pre-gated on presence of CD3.
(TIF)

Figure S2 **A.** resting PBMCs were treated with Gal-9 for 7 days after which cell surface expression of CD14 in adhered monocytic cells was analyzed by flow cytometry. **B.** representative flow cytometric dot-plots of CD4/CD8 staining, **C.** CCR7/CD45RO staining, **D.** CD62L/CD45RO staining. For B-D, cells were pre-gated on presence of CD3.
(TIF)

Figure S3 Representative flow cytometric dot-plots of **A.** CD3/IL-2 staining **B.** CD3/IFNy staining, **C.** CD3/IL-17 staining, **D.** CD3/IL-4 staining.
(TIF)
