# Supplementary figures and images for: Galectin-9 Activates and Expands Human T-Helper 1 Cells
Source: PLoS One. 2013 May 31;8(5):e65616. doi: 10.1371/journal.pone.0065616 (PMC3669208; doi:10.1371/journal.pone.0065616)

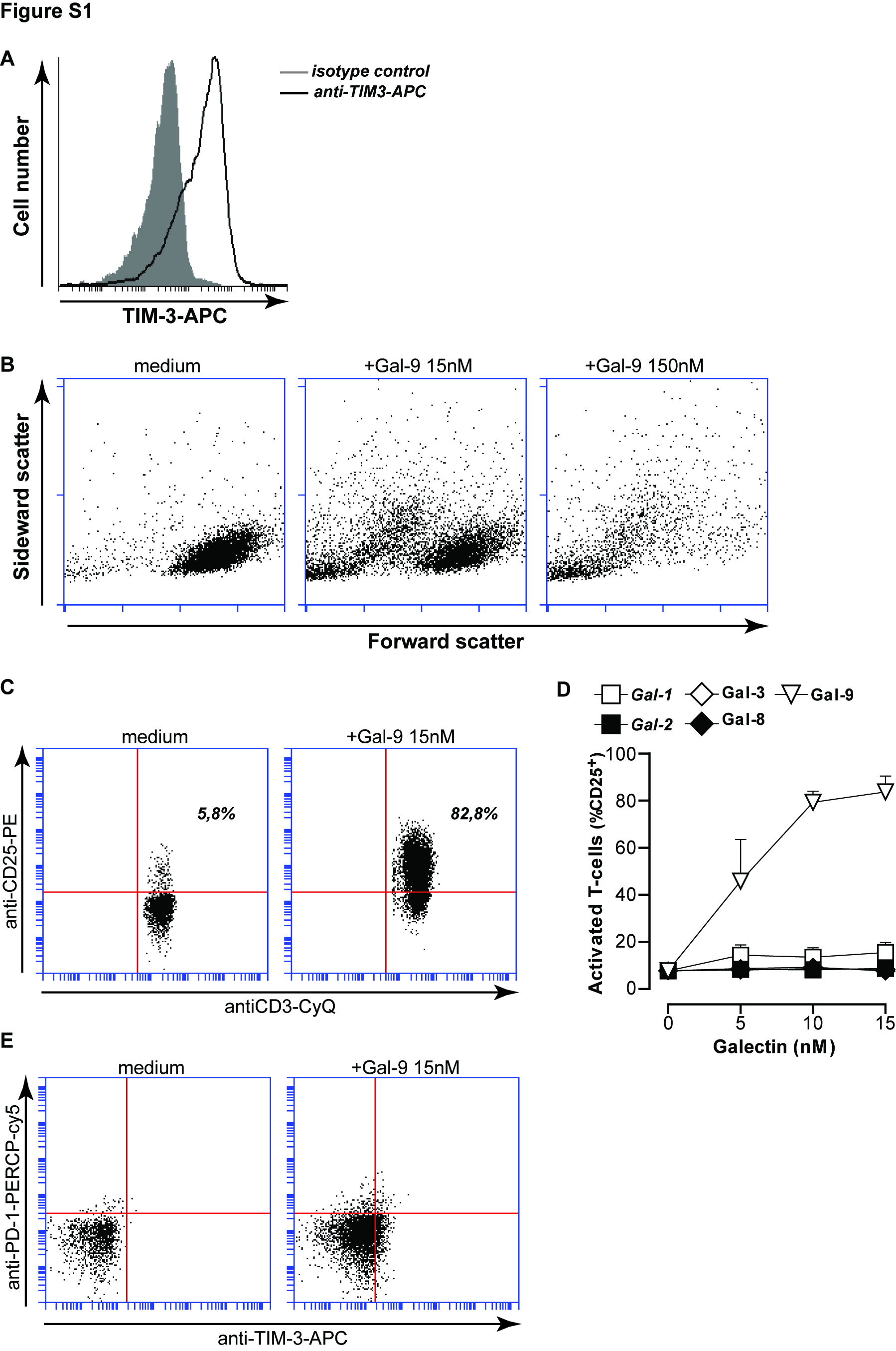

Supplement: Figure S1 — A. T-cells were activated with anti-CD3 (72h) and IL-2 (96h), after which cell surface expression of TIM-3 was analyzed by flow cytometry. B. resting PBMCs were treated with medium, 15 nM or 150 nM of Gal-9 for 1 day. Representative fsc/ssc dot-plots of lymphocytes demonstrate that treatment with 150 nM of Gal-9 shifts cells from a viable population (see medium; left panel) to dead/fragmented distribution (150 nM Gal-9; right panel), whereas at 15 nM ∼50% of cells remain viable (15 nM Gal-9; middle panel). C. representative flow cytometric dot-plots of CD3/CD25 staining D. resting PBMCs were treated with 0-15 nM Gal-1, Gal-2, Gal-3, Gal-8 or Gal-9. E. representative flow cytometric dot-plots of TIM-3/PD-1 staining in which cells were pre-gated on presence of CD3. (TIF) [file pone.0065616.s001.tif]

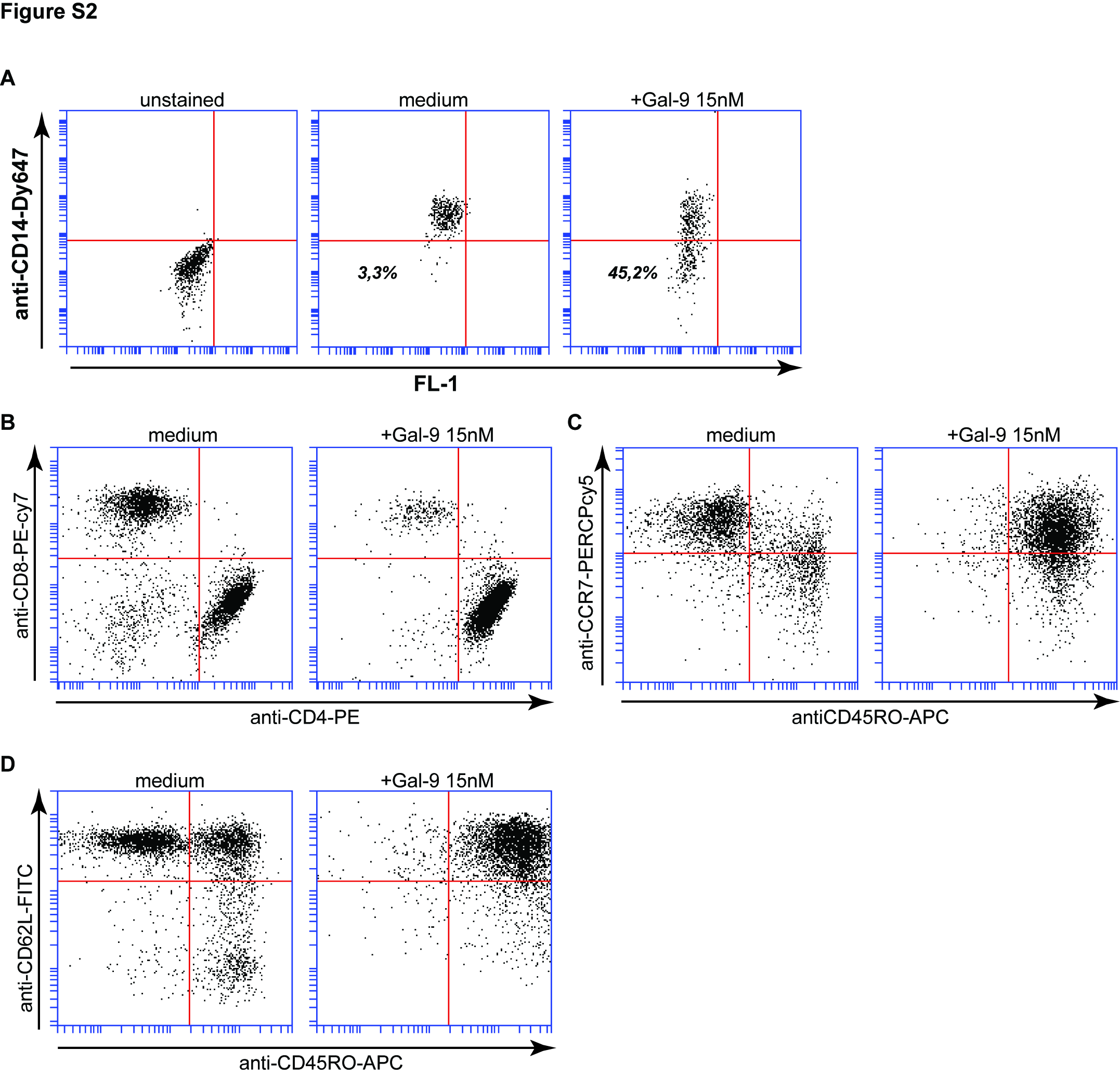

Supplement: Figure S2 — A. resting PBMCs were treated with Gal-9 for 7 days after which cell surface expression of CD14 in adhered monocytic cells was analyzed by flow cytometry. B. representative flow cytometric dot-plots of CD4/CD8 staining, C. CCR7/CD45RO staining, D. CD62L/CD45RO staining. For B-D, cells were pre-gated on presence of CD3. (TIF) [file pone.0065616.s002.tif]

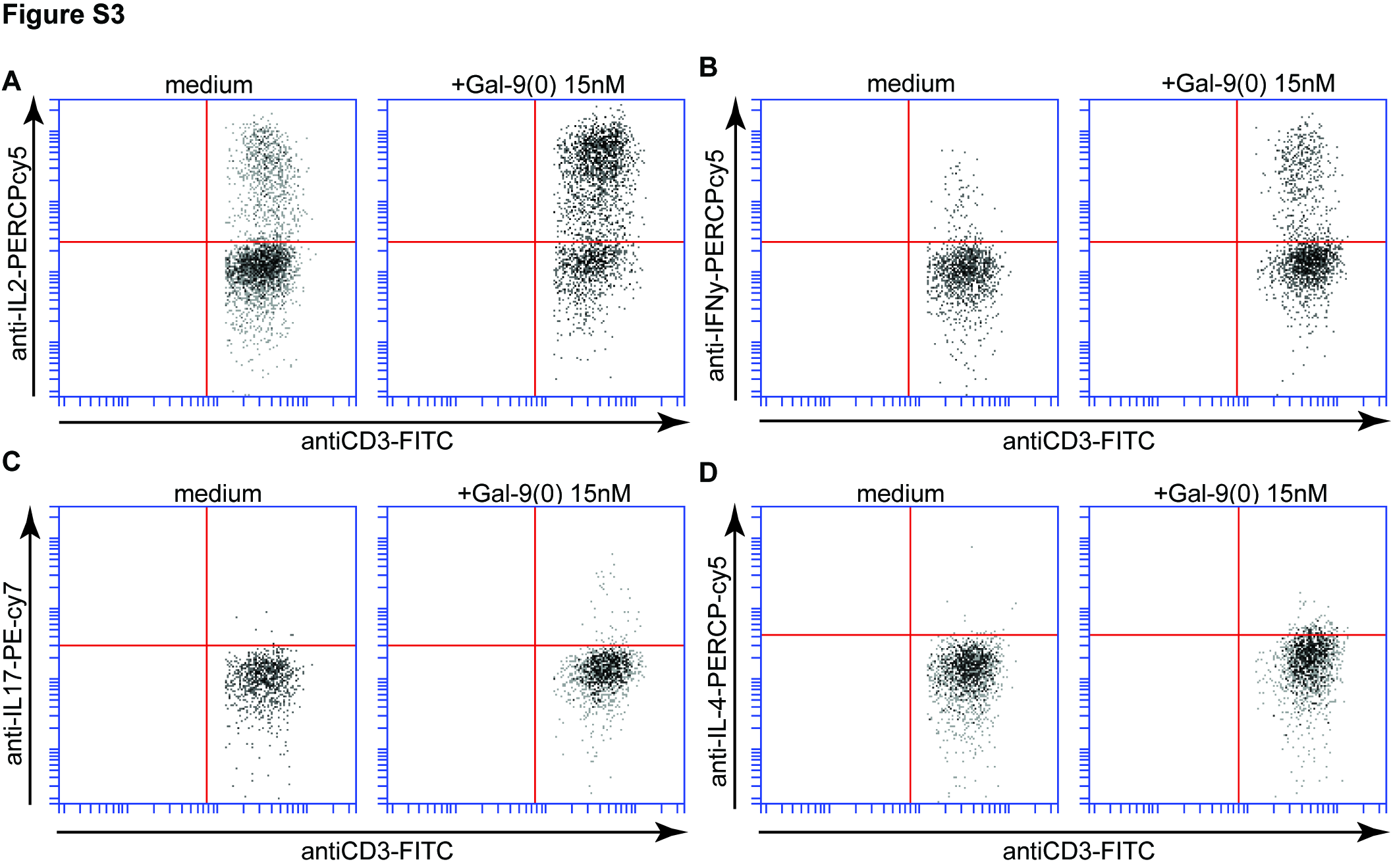

Supplement: Figure S3 — Representative flow cytometric dot-plots of A. CD3/IL-2 staining B. CD3/IFNy staining, C. CD3/IL-17 staining, D. CD3/IL-4 staining. (TIF) [file pone.0065616.s003.tif]
